# A critical perspective of the diverse roles of O-GlcNAc transferase in chromatin

**DOI:** 10.1007/s00412-015-0513-1

**Published:** 2015-04-18

**Authors:** Maria Cristina Gambetta, Jürg Müller

**Affiliations:** MPI of Biochemistry, Chromatin and Chromosome Biology, Am Klopferspitz 18, 82152 Martinsried, Germany

## Abstract

O-linked β-*N*-Acetylglucosamine (O-GlcNAc) is a posttranslational modification that is catalyzed by O-GlcNAc transferase (Ogt) and found on a plethora of nuclear and cytosolic proteins in animals and plants. Studies in different model organisms revealed that while O-GlcNAc is required for selected processes in *Caenorhabditis elegans* and *Drosophila*, it has evolved to become required for cell viability in mice, and this has challenged investigations to identify cellular functions that critically require this modification in mammals. Nevertheless, a principal cellular process that engages O-GlcNAcylation in all of these species is the regulation of gene transcription. Here, we revisit several of the primary experimental observations that led to current models of how O-GlcNAcylation affects gene expression. In particular, we discuss the role of the stable association of Ogt with the transcription factors Hcf1 and Tet, the two main Ogt-interacting proteins in nuclei of mammalian cells. We also critically evaluate the evidence that specific residues on core histones, including serine 112 of histone 2B (H2B-S112), are O-GlcNAcylated in vivo and discuss possible physiological effects of these modifications. Finally, we review our understanding of the role of O-GlcNAcylation in *Drosophila*, where recent studies suggest that the developmental defects in *Ogt* mutants are all caused by lack of O-GlcNAcylation of a single transcriptional regulator, the Polycomb repressor protein Polyhomeotic (Ph). Collectively, this reexamination of the experimental evidence suggests that a number of recently propagated models about the role of O-GlcNAcylation in transcriptional control should be treated cautiously.

## Introduction

### Basics of protein O-GlcNAcylation

O-linked β-*N*-Acetylglucosamine (O-GlcNAc), the monosaccharide modification of serines and threonines in nuclear and cytosolic proteins, was first reported more than 30 years ago (Torres and Hart 1984). O-GlcNAcylation is the only type of glycosylation that occurs in the nucleus and cytosol and is catalyzed by O-GlcNAc transferase (Ogt), using uridine diphosphate (UDP)-GlcNAc as donor of the GlcNAc moiety (Kreppel et al. 1997). Animals contain a single Ogt enzyme and also a single enzyme, O-GlcNAcase (Oga), that removes the modification from nucleocytosolic proteins (Gao et al. 2001).

O-GlcNAc has been proposed to be linked to thousands of proteins that are involved in various distinct cellular processes. Structural studies on Ogt provided insight into how this enzyme modifies this baffling diversity of substrates: Ogt primarily binds to the peptide backbone of substrates and shows no clear specificity for the modification of specific serines or threonines (Janetzko and Walker 2014).

### Biological relevance of O-GlcNAc

Progress towards understanding, the physiological role of O-GlcNAcylation has come from studies on mutant mice, flies, or worms that lack Ogt. Remarkably, Ogt is essential for cell viability in mice, but is required for a specific developmental process in flies, and is dispensable for viability or fertility of worms. This suggests that O-GlcNAcylation has been adopted to participate in one or several essential cellular processes in more complex eukaryotes. In the following, we shall briefly summarize the hallmarks of the *Ogt* mutant phenotypes in these three model organisms.

In mice, Ogt is essential for the viability of embryonic stem cells (Shafi et al. 2000) and all analyzed cell lineages in the developing organism or cultured in vitro (O’Donnell et al. 2004). For example, loss of *Ogt* in T lymphocytes led to apoptosis, and loss of *Ogt* in fibroblasts led to cell growth arrest, senescence, and death, with a failure to undergo four or more cell divisions (O’Donnell et al. 2004). Targeted deletion of *Ogt* in the developing oocyte was also lethal with death occurring at the early postimplantation stage (around day 5 postfertilization) (O’Donnell et al. 2004). Currently, it is not understood why mammalian cells die in the absence of Ogt.

In *Drosophila*, Ogt is not essential for cell viability but it is critically needed for normal development. The fly *Ogt* gene (originally named *super sex combs* but, for simplicity, referred to as *Ogt* in this article) was originally identified as a member of a specific class of transcriptional regulators called the Polycomb group (PcG). These factors, named after their founding member *Polycomb* (Lewis 1978), are required for long-term repression of *HOX* and other developmental regulator genes. Animals lacking *Ogt* arrest development at the end of embryogenesis and display the hallmark phenotype of PcG mutants: characteristic transformations in the body plan arising from a failure to repress transcription of developmental regulator genes in inappropriate cells (Ingham 1984, 1985; Sinclair et al. 2009; Gambetta et al. 2009; Gambetta and Müller 2014). Even though Ogt O-GlcNAcylates many nuclear and cytosolic proteins involved in a wide variety of processes (Kelly and Hart 1989; Sprung et al. 2005; Klement et al. 2010), *Ogt* mutants show no other obvious developmental defects apart from the PcG mutant phenotype (Gambetta and Müller 2014). In addition to its conspicuous role in morphogenesis, *Drosophila Ogt* also participates in physiological processes including circadian rhythm (Kim et al. 2012), glucose-insulin homeostasis (Sekine et al. 2010), and resistance to high temperatures during early stages of embryogenesis (Radermacher et al. 2014).

In stark contrast to mammals and flies, *Caenorhabditis elegans Ogt* null mutants develop into viable adults that show no obvious morphological defects and are fertile (Hanover et al. 2005; Forsythe et al. 2006; Love et al. 2010). Despite the loss of O-GlcNAcylation from many intracellular proteins, the defects in these animals are limited to altered carbohydrate and lipid storage and enhanced insulin-like signaling (Hanover et al. 2005; Forsythe et al. 2006; Love et al. 2010). Ogt thus appears to have a conserved role in the regulation of insulin signaling from worms to man (Hanover et al. 2010).

How is the O-GlcNAc modification removed from proteins? We note that the phenotypes of *Oga* mutants are by far less severe than those of *Ogt* mutants, in both mice (Yang et al. 2012) and flies (Radermacher et al. 2014). *Oga* knockout mice complete embryogenesis but die shortly after birth (Yang et al. 2012) and flies lacking Oga are viable and fertile (Radermacher et al. 2014). One possibility is that protein turnover contributes to the removal of O-GlcNAc. However, it is also possible that, unlike, e.g., phosphorylation or acetylation of proteins, the cycling of O-GlcNAc on and off proteins may simply not always be critical.

### Versatility of O-GlcNAc function

Two fundamental questions in the field are, first, which proteins require O-GlcNAc modification for their function and, second, how does O-GlcNAc alter the properties of modified proteins? In relation to the many O-GlcNAc-modified proteins that have been described, a function for the modification of these proteins has only been reported in a very small fraction, and, on those, a remarkably broad spectrum of molecular mechanisms has been invoked. Depending on the protein, O-GlcNAcylation has been reported to affect its phosphorylation status, enzymatic activity, stability, aggregation, subcellular localization, or association with other proteins or with DNA (reviewed in, e.g., Hart et al. 2007, 2011; Hanover et al. 2012). Moreover, recent studies also unraveled an unsuspected enzymatic activity of Ogt in the proteolytic processing of a specific target protein (Capotosti et al. 2011; Lazarus et al. 2013). Hence, it is not possible to predict how the O-GlcNAc modification affects the molecular properties of a modified protein.

O-GlcNAc is implicated in a bewildering array of basic cellular processes, including signal transduction, cellular differentiation, stress response, and transcriptional regulation (Hart et al. 2007, 2011; Vaidyanathan et al. 2014). Moreover, diverse human diseases such as type II diabetes, Alzheimer’s disease, and cancer have been linked to aberrant O-GlcNAcylation (Ruan et al. 2013; Zhu et al. 2014; Ma and Vosseller 2014). However, because perturbations of global O-GlcNAc levels severely compromise the viability of mammalian cells and lead to pleiotropic effects, ascribing roles of O-GlcNAcylation on a specific protein to the regulation of a specific process remains challenging. One strategy to circumvent this problem is to identify and mutate specific O-GlcNAcylated residues in target proteins to probe the physiological role of the modification. Another line of progress towards unraveling O-GlcNAc function has come from studies in less complex model organisms such as worms and flies.

The levels of protein O-GlcNAcylation in a cell are thought to be directly dependent on the metabolic state of the cell. This is because intracellular UDP-GlcNAc is synthesized through the hexosamine biosynthetic pathway (HBP) in a manner dependent on the availability of glucose, fatty acids, amino acids, and nucleotides. Hence, O-GlcNAcylation is widely believed to also integrate nutrient-dependent cues into O-GlcNAc-regulated processes, a level of regulation that we do not touch upon in this review.

### Functions of O-GlcNAc in the nucleus

Early studies established that Ogt is mainly found within the nucleus and that, quantitatively, most O-GlcNAcylation occurs on nuclear and chromatin-bound proteins (Holt and Hart 1986; Kelly and Hart 1989). The first nuclear protein found to be O-GlcNAcylated is the transcription factor Sp1 (Jackson and Tijan 1988). Since then, proteins involved at each level of transcriptional regulation, including factors regulating DNA methylation, chromatin accessibility, and modification, have been found to be O-GlcNAc modified. There has been a wave of recent reviews that summarize the vast amount of studies that have explored possible mechanisms of how Ogt regulates transcription (e.g., Ozcan et al. 2010; Hanover et al. 2012; Lewis 2013; Gut and Verdin 2013; Vaidyanathan et al. 2014; Jóźwiak et al. 2014; Forma et al. 2014; Dehennaut et al. 2014; Lewis and Hanover 2014; Harwood and Hanover 2014). Here, we critically assess the methodologies and original evidence that served as basis for establishing current views on how O-GlcNAcylation might impart on transcription. We discuss these findings in the context of *Ogt* mutant phenotypes in vertebrates and invertebrates.

## The evidence for a role of Ogt in transcriptional regulation

Several lines of evidence suggest that gene transcription is a major process that is regulated by O-GlcNAcylation. (i) Genome-wide profiling of O-GlcNAc or Ogt by chromatin immunoprecipitation (ChIP) assays found that O-GlcNAcylated proteins or Ogt bind at specific chromosomal sites in worms, flies, and mammals. (ii) Biochemical purifications from mammalian cells revealed that Ogt stably associates with several transcriptional regulators. (iii) Proteins acting at all steps of gene transcription, including histones, have been reported to be O-GlcNAcylated. (iv) Mechanistic and genetic studies demonstrate that O-GlcNAcylation of a subset of these transcription regulators is critical for their function.

### Genome-wide profiling of O-GlcNAcylation and Ogt

Different antibodies have been used to monitor the chromosomal localization of O-GlcNAc (Table 1). In flies and worms, the absence of O-GlcNAc signals in *Ogt* null mutant animals has provided direct proof that these profiles indeed represent the binding of O-GlcNAcylated transcription factors and/or other chromatin-associated proteins. In mammalian cells, this type of control is obviously less straightforward because of the deleterious effects caused by depletion of Ogt, a point that should be kept in mind when interpreting O-GlcNAc profiles generated in mammalian cells. In addition to O-GlcNAc, Ogt itself has been reported to be present at specific chromosomal sites in mammalian cells (Table 1). Whether Ogt also associates with specific chromosomal sites in flies or worms is currently not known and awaits further investigation. In the following, we discuss our current understanding of what the reported O-GlcNAc profiles represent in the different species.

**Table 1:**
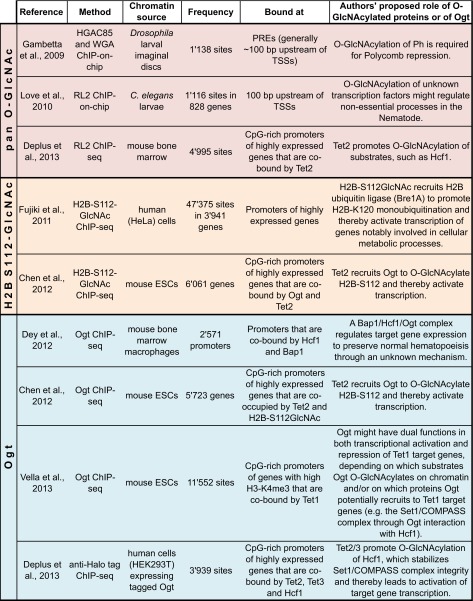
Published genome-wide distributions of Ogt and O-GlcNAcylated proteins

O-GlcNAcylated proteins have been profiled in flies, worms, and mammals using either pan O-GlcNAc antibodies RL2 (described in Holt et al. 1987) or HGAC85 (described in Turner et al. 1990) or using the lectin wheat germ agglutinin (WGA). Examples of ChIP-seq profiles obtained using a monoclonal antibody raised against a synthetic histone H2B peptide GlcNAcylated on serine 112 (Fujiki et al. 2011) are shown in Fig. 2 (see text for details). Direct binding of Ogt itself to chromatin has thus far only been reported in mammalian cells but not in flies or worms

#### Drosophila

Early studies using wheat germ agglutinin (WGA), a lectin that binds to O-GlcNAc, first reported that the modification is widely distributed on *Drosophila* chromosomes (Kelly and Hart 1989). More recent genome-wide O-GlcNAc profiling studies in *Drosophila* revealed that the modification is highly enriched at sites bound by PcG repressor proteins (Gambetta et al. 2009) (Table 1). In flies, PcG proteins bind discrete nucleosome-depleted regions termed Polycomb response elements (PREs), generally found near transcription start sites (TSSs) of respective target genes (Oktaba et al. 2008). The observation of O-GlcNAc at PcG protein binding sites goes hand in hand with the finding that Ogt O-GlcNAcylates one of the PcG repressor proteins, Polyhomeotic (Ph), and that this is critical for Polycomb repression (Gambetta et al. 2009; Gambetta and Müller 2014). Nevertheless, Ph is not the only Ogt substrate and several other chromatin-bound proteins have been reported to be O-GlcNAcylated in *Drosophila* (Holt and Hart 1986; Kelly and Hart 1989).

#### *C. elegans*

In worms, O-GlcNAcylated proteins are present at discrete locations near TSSs of various genes (Love et al. 2010) (Table 1). The identities of the detected O-GlcNAcylated protein(s) are currently not known (Love et al. 2010). The finding that the level of O-GlcNAc signals in the chromatin from *Oga* mutants was increased suggests an active cycling of the modification at target genes in wild-type animals (Love et al. 2010). Considering that Ogt is not essential for development and morphogenesis of *C. elegans* under standard physiological conditions, the function of this chromatin-associated O-GlcNAcylation still remains to be elucidated.

#### Mammalian cells

In mammalian cells, both Ogt and O-GlcNAcylated substrates were detected at TSSs of CpG-rich promoters of actively transcribed genes (Chen et al. 2012; Deplus et al. 2013; Vella et al. 2013) (Table 1). Moreover, the profile of an O-GlcNAc-modified form of histone H2B (H2B-S112GlcNAc) has been reported (Fujiki et al. 2011; Chen et al. 2012) and an assessment of these data is presented further below. A general conclusion that has been put forward is that in mammals, unlike in *Drosophila*, O-GlcNAcylation of chromatin-bound proteins is linked to transcriptional activation (Table 1). Notwithstanding, Ogt was also found to O-GlcNAcylate many proteins involved in transcriptional repression (e.g., Ozcan et al., 2010); O-GlcNAcylated repressor proteins bound to chromatin are thus also expected to contribute to the signals in pan-O-GlcNAc genome-wide profiling studies.

In the following, we address two main questions that arise from the observation that Ogt can associate with chromatin in mammals: Why is Ogt localized at specific chromosomal sites, and is there a function of chromatin-bound Ogt?

### Ogt stably associates with specific transcription factors

Unbiased Ogt protein purifications have thus far only been performed in mammalian cells and have identified Hcf1 and Ten-eleven translocation (Tet) proteins as prominent Ogt interactors. Ogt-Hcf1 and Ogt-Tet complexes are distinct (Vella et al. 2013; Deplus et al. 2013), and thus interaction of Ogt with Tet proteins or Hcf1 may be two major routes to recruit Ogt to specific chromosomal loci in mammals. In the case of Tet proteins, a role in the recruitment of Ogt to chromatin has indeed recently been demonstrated (see below). This has led to the hypothesis that Ogt recruitment to specific chromosomal sites might be a prerequisite for localized O-GlcNAcylation of substrates that require this modification in order to execute their function in transcription (Chen et al. 2012; Vella et al. 2013; Deplus et al. 2013). In the following, we critically evaluate this hypothesis.

#### The Ogt-Hcf1 interaction

Biochemical purification of Ogt from nuclear extracts of mammalian cells identified the conserved Hcf1 transcriptional co-regulator as the most abundant Ogt interactor in two independent Ogt purifications (Vella et al. 2013; Deplus et al. 2013) (Table 2). Conversely, Ogt was found to co-purify with Hcf1 from mammalian cells (Wysocka et al. 2003) (Table 2). It has been suggested that approximately 50 % of nuclear Ogt is stably associated with Hcf1 in HeLa cells and that Hcf1 stabilizes Ogt protein levels and impacts on its nuclear localization (Daou et al. 2011; Ruan et al. 2012). In addition, Ogt and Hcf1 were found to co-purify together with numerous other nuclear protein complexes (Table 2). Hcf1 has been described to interact with a plethora of transcriptional regulators (reviewed in Wysocka and Herr 2003; Zargar and Tyagi 2012) and, in two cases, Ogt association with these complexes has been demonstrated to indeed be mediated by Hcf1 (Mazars et al. 2010; Ruan et al. 2012). Consistent with the stable Hcf1-Ogt interaction, chromosomal binding sites of Hcf1 and Ogt overlapped in mouse (Dey et al. 2012) and human cells (Deplus et al. 2013) (Table 1). The interaction between Hcf1 and Ogt occurs in part through the binding of threonine-rich regions present in the central portion of Hcf1 to a highly conserved ladder of asparagine residues present in the tetratricopeptide repeats (TPR) in Ogt, an interaction that was recently visualized in a crystal structure (Lazarus et al. 2013). Ogt associates in addition with the N-terminal portion of Hcf1 (Wysocka et al. 2003; Daou et al. 2011).

**Table 2:**
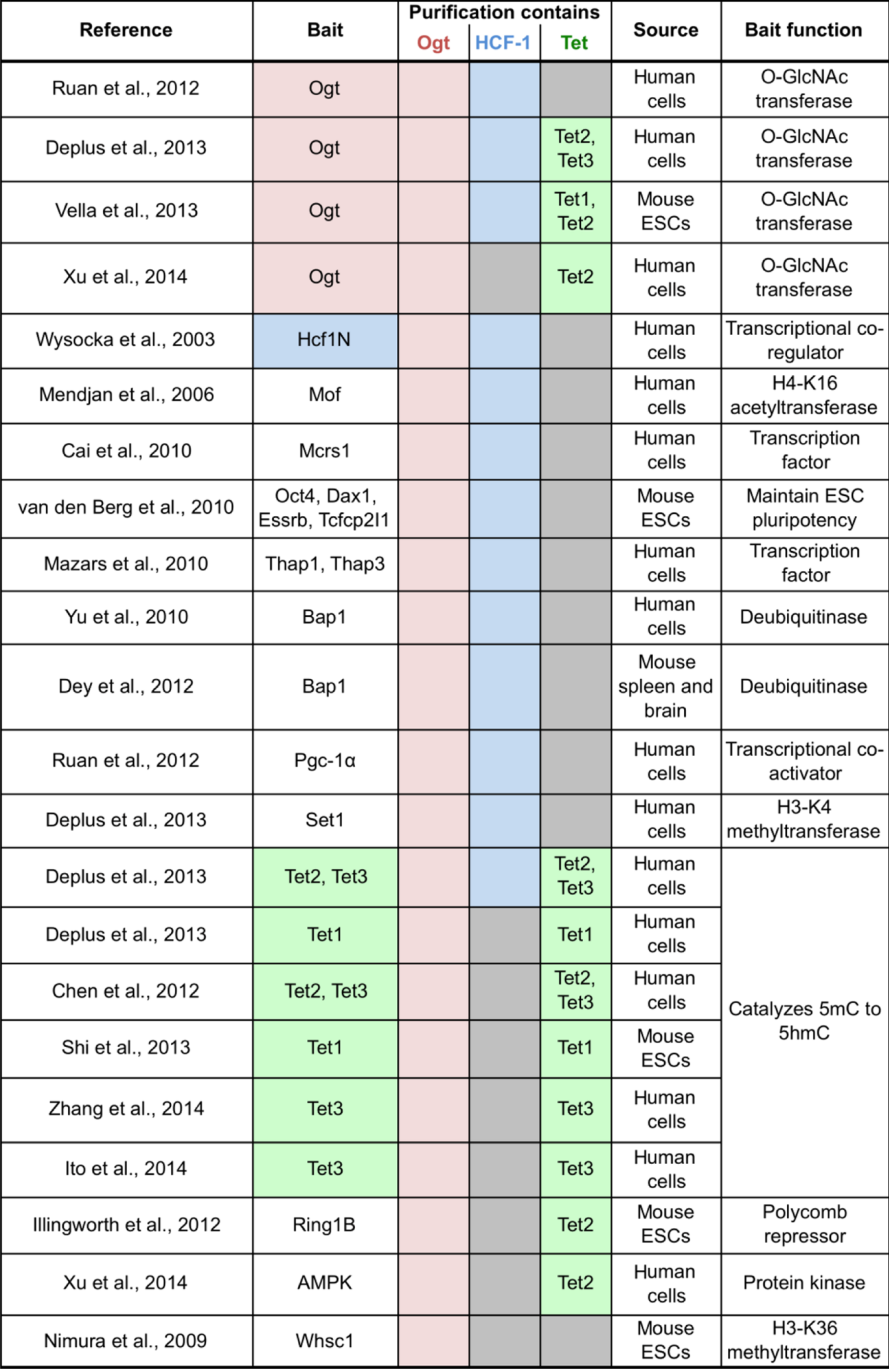
Published protein purifications containing Ogt

Ogt has been co-purified with numerous nuclear proteins from mammalian cells. Of interest, Ogt directly and stably interacts with the Hcf1 and Tet transcription factors (reported recoveries of Ogt, Hcf1, or Tet proteins in each purification are shaded in pink, blue, or green, respectively; gray boxes indicate that Hcf1 or Tet proteins were not reported to be recovered). Hcf1 likely bridges Ogt to several transcription factors with which Hcf1 associates. Note that whereas Ogt and Hcf1 frequently co-purify in a variety of different protein assemblies, Tet-Ogt interactions are primarily recovered in purifications of Ogt and Tet proteins but they mostly do not co-purify in the context of other protein assemblies

What is the function of the Ogt-Hcf1 interaction? The possibly best understood function comes from the unexpected discovery that Ogt is the enzyme responsible for the proteolytic maturation of Hcf1 in mammalian cells (Capotosti et al. 2011). Hcf1 is synthesized as a large precursor protein that is subsequently cleaved at a series of six centrally located repeats, generating two N- and C-terminal subunits that remain stably associated (Wilson et al. 1995) and that play a major function in regulating different aspects of the cell cycle (Julien and Herr 2003). Subsequent structural studies revealed that Hcf1 GlcNAcylation and proteolytic cleavage occur in the same active site and that the cleavage reaction uses UDP-GlcNAc as a co-substrate for the reaction mechanism (Lazarus et al. 2013). Hcf1 is also highly O-GlcNAcylated (Wilson et al. 1993; Capotosti et al. 2011) and has been identified in diverse mass spectrometry studies aimed at mapping O-GlcNAcylation sites in the proteome (Wells et al. 2002; Khidekel et al. 2004, 2007; Wang et al. 2010a, b; Hahne et al. 2012). Ogt-induced cleavage is required for proper M phase progression; if the Hcf1 central proteolytic repeats are replaced by sites for a heterologous protease, Hcf1 is processed yet cells become binucleated (Capotosti et al. 2011). Intriguingly, in *Drosophila*, Hcf1 is proteolytically processed by a different protease called Taspase 1 (Capotosti et al. 2007) but, nevertheless, it is also O-GlcNAcylated by Ogt (Gambetta and Müller 2014). In the absence of O-GlcNAcylation, *Drosophila* Hcf1 forms large molecular weight aggregates (Gambetta and Müller 2014). An important function of Hcf1 O-GlcNAcylation might be to prevent Hcf1 aggregation, as discussed for another protein in the last section of this article.

Because of the extensive genome-wide co-binding of Ogt and Hcf1, one might posit that the recruitment of Ogt to chromatin by Hcf1-containing complexes might be functionally important for O-GlcNAcylation of other substrates. However, a simple explanation for Ogt and Hcf1 co-localization might be that it simply reflects the stable association of Hcf1 with Ogt during proteolytic processing of Hcf1.

#### The Ogt-Tet interaction

Other abundant Ogt interactors are Tet enzymes (Vella et al. 2013; Deplus et al. 2013) (Table 2). Reciprocally, several independent studies identified Ogt as the major interactor of Tet proteins in mouse embryonic stem cells (ESCs) and in human cells (Chen et al. 2012; Deplus et al. 2013; Vella et al. 2013; Shi et al. 2013; Ito et al. 2014; Zhang et al. 2014) (Table 2). Tet proteins directly interact with Ogt through their C-terminal catalytic domains (Chen et al. 2012; Deplus et al. 2013). Genome-wide binding studies in mouse and human cells suggest that Ogt-bound sites extensively overlap with Tet-bound sites (Chen et al. 2012; Vella et al. 2013; Deplus et al. 2013) (Table 1).

What is the function of the Ogt-Tet interaction? Studies in mouse ESCs depleted of Tet proteins suggest that these proteins are responsible for targeting about 50 % of chromatin-associated Ogt (Chen et al. 2012; Vella et al. 2013). Different laboratories have proposed different proteins as substrates that are then O-GlcNAcylated by Tet-tethered Ogt: H2B-S112 (Chen et al. 2012), Hcf1 (Deplus et al. 2013), or other transcriptional regulators (Vella et al. 2013). Specifically, Chen et al. (2012) proposed that transcriptional activation is achieved through O-GlcNAcylation of H2B-S112—a poorly characterized modification whose existence is controversial (see next section). Deplus et al. (2013), in contrast, proposed that Tet-tethered Ogt O-GlcNAcylates Hcf1, a process that the authors surprisingly claimed to be required for integrity of the H3-K4 methyltransferase complex Set1/COMPASS. However, all these models pose a major conundrum: *Ogt* KO mice are early embryonic lethal whereas *tet1*/*tet2* double KO mice are viable and fertile (Dawlaty et al. 2013) and *tet3* KO mice survive until after birth (Gu et al. 2011). This raises questions about the significance of Ogt targeting by Tet proteins to specific chromosomal sites.

Conversely, Ogt is not required for the recruitment of Tet proteins to chromatin (Chen et al. 2012; Ito et al. 2014). Tet has been found to be O-GlcNAcylated by Ogt (Myers et al. 2011; Vella et al. 2013; Shi et al. 2013; Zhang et al. 2014), but the function of O-GlcNAcylation of Tet proteins is not yet understood. Tet proteins are believed to regulate gene transcription notably through the enzymatic oxidation of the repressive cytosine DNA methylation mark (5mC) into hydroxymethylcytosine (5hmC) (Pastor et al. 2013); however, the enzymatic activity of Tet proteins is unaffected in the absence of O-GlcNAcylation (Chen et al. 2012; Deplus et al. 2013; Ito et al. 2014).

### O-GlcNAcylation of histones: assessing the evidence

Posttranslational modifications on histone proteins, notably on their N-terminal tails, impact on transcription by marking nucleosomes for interaction with chromatin-binding proteins or by directly affecting chromatin structure (e.g., Hecht et al. 1996; Braunstein et al. 1993; Pengelly et al. 2013; reviewed in e.g., Shahbazian and Grunstein 2007; Bannister and Kouzarides 2011). Recent studies have reported that mammalian histones are also O-GlcNAcylated (Sakabe et al. 2010; Zhang et al. 2011; Fujiki et al. 2011; Schouppe et al. 2011; Fong et al. 2012; Hahne et al. 2012) (Table 3). In the following, we evaluate the methodology and the actual data that served as the foundation for the conclusion of these studies.

**Table 3:**
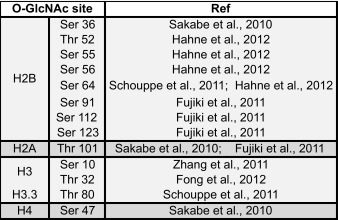
List of reported O-GlcNAcylated sites on vertebrate core histones

The direct identification of O-GlcNAcylated serines or threonines by tandem mass spectrometry is challenging. For example, conventional peptide fragmentation by collision-induced dissociation (CID) frequently results in cleavage of the highly labile glycosidic bond and loss of the GlcNAc moiety. An alternative fragmentation approach called electron transfer dissociation (ETD) is the method of choice for accurate O-GlcNAc site localization by mass spectrometry because it robustly fragments the peptide backbone while retaining the modification on the hydroxyl amino acid, thus enabling direct mapping of the modified amino acid (Khidekel et al. 2007). Among all O-GlcNAcylated histone residues reported, only one, Thr32 on histone H3 (H3-T32), was identified by this approach (Fong et al. 2012) (Table 4). It is important to note that none of the other studies that analyzed histones isolated from cells actually directly identified the GlcNAc modification by mass spectrometry (Sakabe et al. 2010; Zhang et al. 2011; Fujiki et al. 2011; Schouppe et al. 2011) (Table 4). Specifically, Sakabe et al. (2010) and Schouppe et al. (2011) relied on an elaborate indirect strategy in which all posttranslational modifications, including O-GlcNAcylation and also phosphorylation, were released from serines and threonines and substituted by stable adducts that resist CID-type fragmentation (Wells et al. 2002). Zhang et al. (2011) relied entirely on detection of O-GlcNAc on histones by western blot analysis using anti-O-GlcNAc antibodies. In yet another strategy, Fujiki et al. (2011) reacted free histones with Ogt and UDP-GlcNAc in a 24-h reaction in vitro, raised an antibody against a synthetic GlcNAcylated H2B-S112 (H2B-S112GlcNAc) peptide—one of the O-GlcNAc-modified histone residues that they found by this approach—and then used this antibody for all subsequent experiments in their study.

**Table 4:**
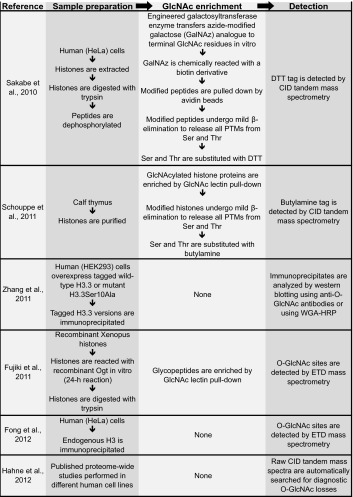
Published strategies that have been used to map O-GlcNAc sites on vertebrate histones

A wide variety of strategies have been used to identify potential O-GlcNAc sites on vertebrate histones. The principal steps of histone sample preparation, followed by enrichment and mapping of these O-GlcNAc sites, are summarized in this table [see Ma and Hart (2014) for a review on general O-GlcNAc mapping strategies]. The inherent lability of O-GlcNAc during CID-type peptide fragmentation complicates direct and straightforward identification of O-GlcNAcylated histone residues. The mild β-elimination strategies used by Sakabe et al. and Schouppe et al. were combined with controls in order to establish that the reacted peptide originally contained O-GlcNAc and not phosphate. Although not ideal for O-GlcNAc site localization, the initial detection of O-GlcNAc peptides can be facilitated in CID-type experiments because diagnostic O-GlcNAc losses define a characteristic pattern that can identify O-GlcNAcylated peptides in complex proteomic samples—a strategy that was used by Hahne et al. (2012) to report O-GlcNAc sites on H2B

*DTT* dithiothreitol, *PTM* posttranslational modification, *CID* collision-induced dissociation, *ETD* electron-transfer dissociation, *HRP* horse radish peroxidase

**Fig. 1:**
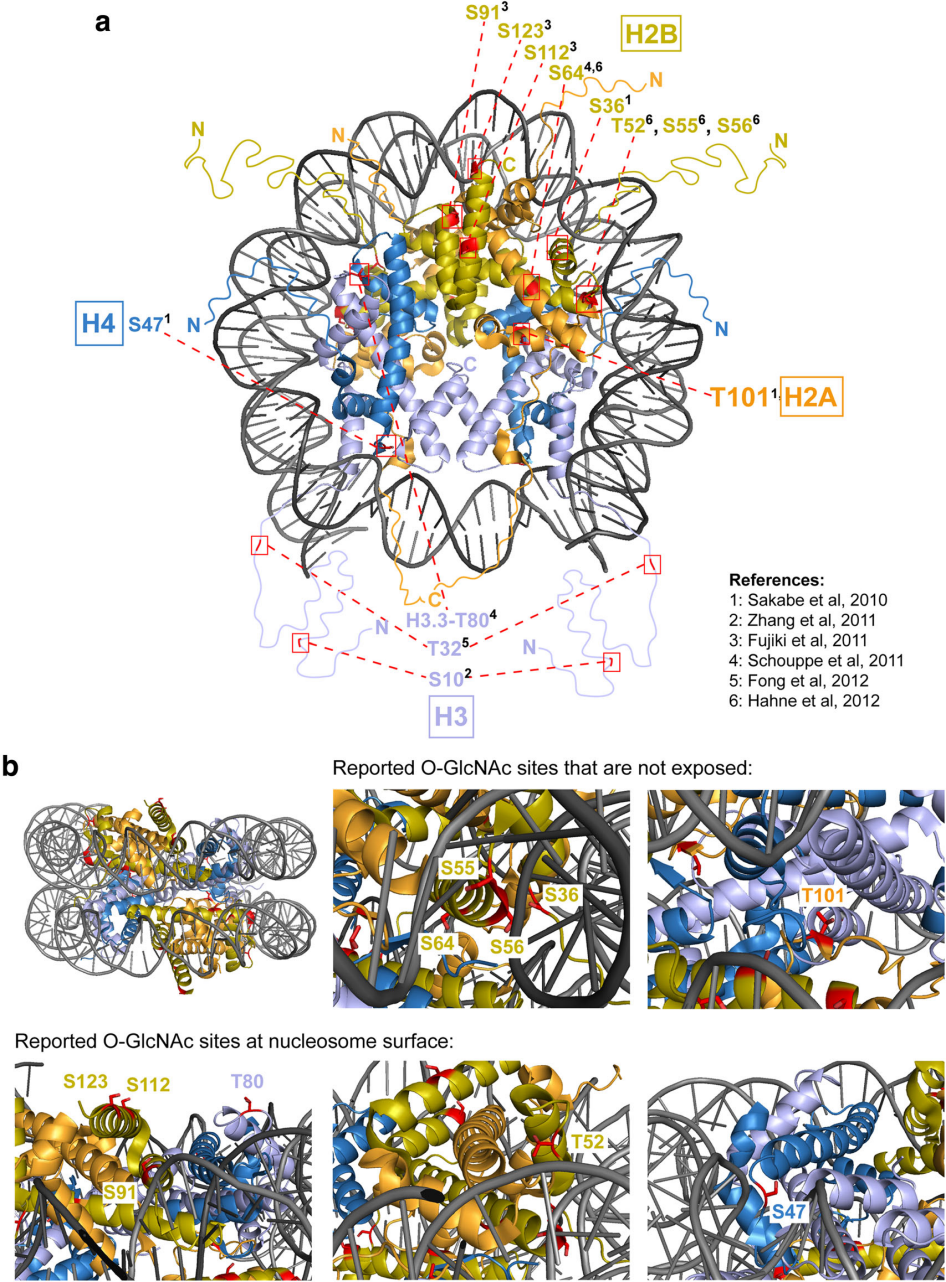
Reported O-GlcNAcylated residues in histone proteins mapped onto the nucleosome structure. **a** Top view of the human nucleosome crystal structure (PDB 3AFA, Tachiwana et al. 2010). The nucleosome is the basic unit of chromatin and is composed of a tetramer of histones H3 and H4 and two H2A-H2B dimers around which 147 bp of DNA is wrapped. N- and C-terminal unstructured extensions are schematized and labeled. Serines or threonines of canonical histones that have been proposed to be O-GlcNAc modified in vivo are highlighted in *red* (summarized in Table 3). **b** Lateral view of the nucleosome. Zoomed-in views of candidate O-GlcNAcylated histone residues predicted either to be inaccessible to Ogt in the context of the assembled nucleosome (*top row*) or accessible because exposed at the nucleosome surface (*bottom row*)

These studies taken together raise three important issues. First, each of these studies identified a different set of residues, and only two of the 13 identified sites were identified in two independent studies (Table 3). Second, half of the reported O-GlcNAc sites are buried in histone-histone or histone-DNA interfaces and therefore inaccessible to Ogt in the context of the assembled nucleosome (Fig. 1). Hence, O-GlcNAcylation at most of these sites on histones would have to occur before these assemble into nucleosomes. Third, if histones become O-GlcNAcylated in a non-nucleosome context, this raises the question to what extent the O-GlcNAc-modified histones would be able to become incorporated into nucleosomes. O-GlcNAcylation thus remains an enigmatic histone modification.

**Fig. 2:**
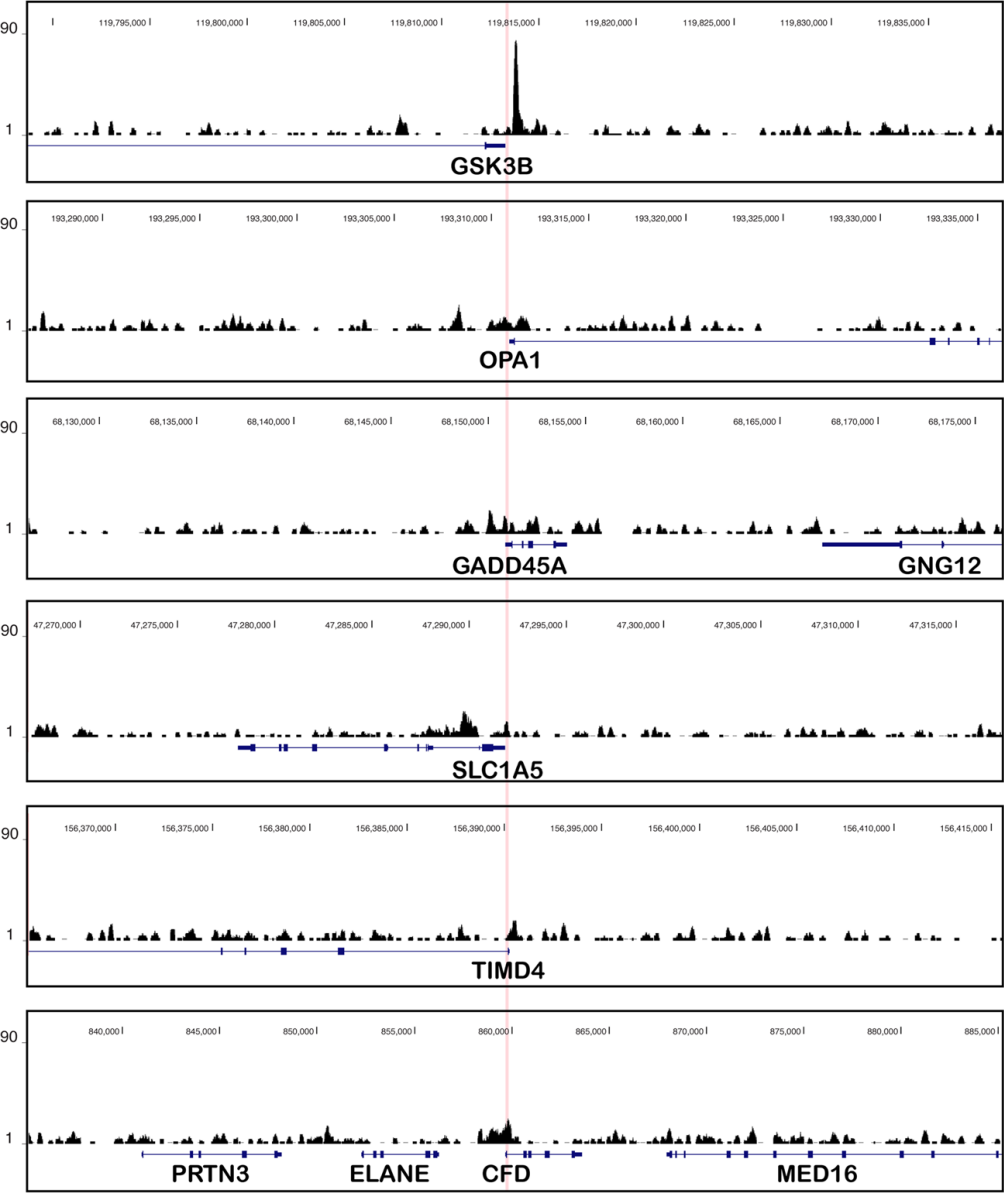
Published ChIP-seq profiles of H2B-S112GlcNAc in human cells. Published ChIP-seq profiles (Fujiki et al. 2011) at six genes for which the authors reported significant H2B-S112GlcNAc enrichment near their TSSs in HeLa cells. The profile at GSK3B was reported in Fig. 4e of Fujiki et al (2011); the five other genes were randomly chosen among the first 20 genes at the top of the list showing H2B-S112GlcNAc enrichment in Table S3A (Fujiki et al 2011). The ChIP-seq profiles are centered on each TSS (highlighted in *pink*) and extend 25 kb upstream and downstream (50 kb windows are shown in total), RefSeq genes are indicated with exons (*boxes*) and introns (*thin lines*), and genome coordinates are indicated *above* (version hg19). For all profiles, we used the same scale on the *y*-axis. Note that unlike the enrichment of H2B O-GlcNAcylation ChIP signal at GSK3B, the signals at the other positively scored genes are approaching background ChIP signals

Recently, Fujiki et al. (2011) and Chen et al. (2012) reported the genome-wide distribution of H2B-S112GlcNAc. The authors described that H2B-S112GlcNAc is enriched at TSSs of highly expressed genes (Table 1). However, our inspection of the reported H2B-S112GlcNAc ChIP-seq profile in HeLa cells (Fujiki et al. 2011) reveals that these profiles might not be of sufficient quality to justify conclusions about specifically enriched regions (Fig. 2).

### The role of Ogt in Polycomb repression

The models discussed above posit that the function of Ogt in transcriptional regulation relies on it being tethered to chromatin to modify its substrates. Nevertheless, Ogt is also able to modify transcription factors to which it does not stably bind and it may likely also modify such factors off chromatin. The finding that Ogt is essential for Polycomb-mediated gene repression in *Drosophila* constitutes one of the best-characterized examples of a role of Ogt in transcriptional regulation.

PcG proteins assemble into multiprotein complexes including the Polycomb Repressive Complexes 1 and 2 (PRC1 and PRC2) which harbor enzymatic activities for the covalent modification of histones (reviewed in e.g., Beisel and Paro 2011; Simon and Kingston 2013). These complexes bind to their target genes to repress transcription through histone modification and through the compaction of chromatin (Beisel and Paro 2011; Simon and Kingston 2013). Recent biochemical and genetic analyses in *Drosophila* unraveled the molecular mechanism through which Ogt contributes to Polycomb repression in flies. Below, we critically review reports on the role of Ogt in Polycomb repression in both flies and mammals.

#### O-GlcNAcylation of Polyhomeotic—a key function of Ogt in flies

**Fig. 3:**
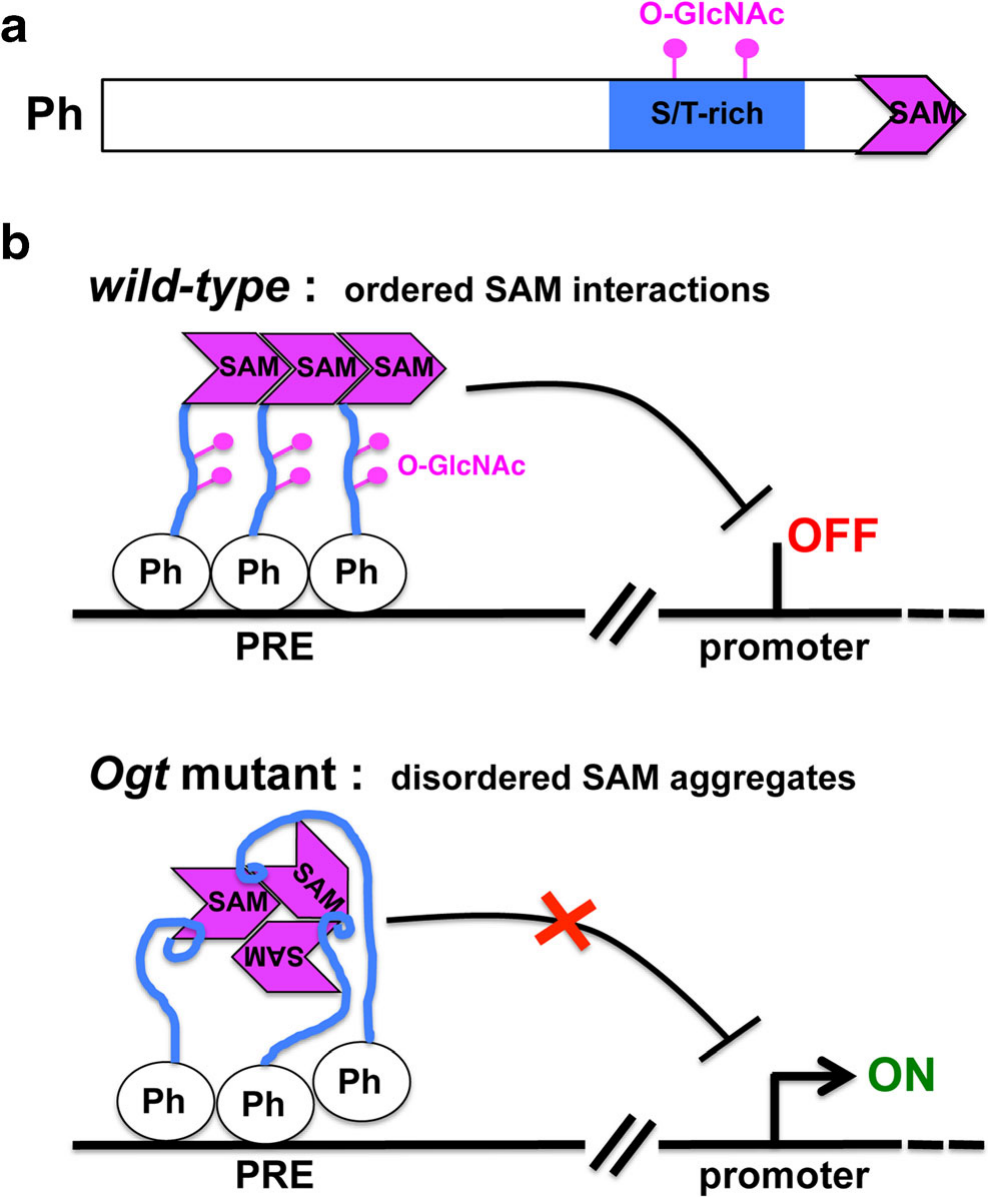
Model of O-GlcNAcylation function in Polycomb repression in *Drosophila*. **a** Schematic representation of the fly Ph protein that is O-GlcNAcylated on an S/T-rich region, located close to the C-terminal SAM domain. **b** Model illustrating how O-GlcNAcylation of Ph allows the formation of ordered SAM-SAM assemblies that are needed to silence Polycomb target genes (*top*). Ph is bound at PREs as part of the PRC1 complex (other PRC1 subunits are not shown) in both wild-type and *Ogt* mutant animals. In the absence of O-GlcNAcylation of the S/T stretch, Ph molecules aggregate through their SAM domains (Gambetta and Müller 2014) (*bottom*). The exact molecular mechanism through which O-GlcNAcylation of the S/T-rich stretch prevents Ph molecules from engaging in non-productive contacts with other SAM domains is not known, but it might involve intramolecular contacts (illustrated here as small loops) between the S/T stretch and the SAM domain that alter SAM conformation in a way that favors aggregation with other SAM domains in a similar conformation (Gambetta and Müller 2014)

Ogt does not stably associate with any of the other PcG proteins in *Drosophila*, but it specifically O-GlcNAcylates one of the PcG proteins, the PRC1 subunit Polyhomeotic (Ph) (Gambetta et al. 2009). Like Ph, the O-GlcNAc modification is highly enriched at PREs (Gambetta et al. 2009; Sinclair et al. 2009) (Table 1). In the absence of O-GlcNAcylation—in *Ogt* mutants—Ph still incorporates into PRC1 and is bound at PREs (Gambetta et al. 2009; Gambetta and Müller 2014). However, these PRE-bound Ph assemblies are defective; they form large soluble aggregates upon extraction from chromatin (Gambetta and Müller 2014). Ph was found to be highly GlcNAcylated on a serine/threonine (S/T)-rich region, and this modification prevented the aggregation of Ph molecules in vitro (Gambetta and Müller 2014) (Fig. 3). *Drosophila* mutants expressing a Ph protein that lacks the O-GlcNAcylated S/T stretch precisely reproduce the phenotype of *Ogt* mutants (Gambetta and Müller 2014). The key function of Ogt during fly development thus appears to be to O-GlcNAcylate the S/T-rich region in Ph.

Ph represses Polycomb target genes through its Sterile Alpha Motif (SAM) domain that engages in ordered interactions with the SAM domain in other Ph molecules or in Scm, another PRC1 subunit (Kim et al. 2002, 2005; Robinson et al. 2012; Isono et al. 2013; Gambetta and Müller 2014). In Ph molecules in which the S/T-rich region is not O-GlcNAcylated, the SAM domain is unable to form ordered assemblies (Gambetta and Müller 2014). This likely explains why repression of Polycomb target genes is defective in *Ogt* mutant animals (Fig. 3). Intriguingly, human Ph homologs also require O-GlcNAcylation to prevent aggregation through their SAM domains (Gambetta and Müller 2014), raising the possibility that this function of Ogt in Polycomb repression is conserved in mammals—an idea that yet has to be investigated. Similarly, it is currently not known whether O-GlcNAcylation of Ph has evolved as a means to modulate Ph repressor function, or whether evolution has applied it to maintain Ph dispersity.

#### Do Ogt and PRC2 regulate each other?

PRC2 is a histone methyltransferase that catalyzes trimethylation of H3-K27 to repress genes in animals (Laugesen and Helin 2014). A recent study found that Ogt stabilizes the PcG protein complex PRC2 in a specific human breast cancer cell line (Chu et al. 2014). Knock-down of Ogt in this cell line reduced PRC2 levels and decreased bulk H3-K27me3 levels by approximately 50 % (Chu et al. 2014). EZH2, the catalytic subunit of PRC2, was found to be O-GlcNAcylated on Ser75, a residue proposed to be critical for EZH2 protein stability (Chu et al. 2014). This led to the suggestion that O-GlcNAcylation of Ser75 stabilizes the EZH2 protein and thereby permits effective H3-K27 trimethylation at and repression of selected target genes (Chu et al. 2014). In contrast to these observations in breast cancer cells, knock-down of Ogt in mouse ESCs did not result in a detectable reduction of EZH2 or H3-K27me3 levels (Myers et al. 2011). Levels of PRC2 and H3-K27me3 were also found to be unperturbed in *Ogt* mutant *Drosophila* (Gambetta et al. 2009). It therefore remains to be determined whether the observed destabilization of EZH2 upon Ogt knock-down is restricted to particular cell lines in mammals.

A previous study also proposed the reverse regulatory relationship: PRC2 was reported to be required for normal Ogt protein and O-GlcNAc levels (Myers et al. 2011). Specifically, mouse ESC lines lacking the PRC2 core subunits Eed or Suz12 showed reduced Ogt and O-GlcNAc levels (Myers et al. 2011). The reason for this is currently unclear. This study did not provide any evidence that Ogt would contribute to Polycomb repression in mammals, but we caution that it is nevertheless frequently referred to for doing so.

## Conclusions

One of the most remarkable properties of the O-GlcNAc modification is that it is present on such a stunningly large number of proteins in worms, flies, and mammals but that removal of this modification has so vastly different consequences in these organisms. A provocative thought therefore is that on the majority of O-GlcNAcylated proteins, the modification may have little or no function. Therefore, the identification of substrates on which the O-GlcNAc modification is indeed critically needed for in vivo function and deciphering how O-GlcNAc alters the molecular properties of these modified proteins are key tasks to pin down the physiologically relevant mechanisms of this modification. Here, we discussed our current understanding of the role of Ogt and O-GlcNAcylation in Hcf1 processing and maturation, and recent progress that elucidated how O-GlcNAcylation of Polyhomeotic impacts on Polycomb repression, two processes where the role of Ogt and O-GlcNAcylation is well supported by in vivo functional data. Our review of the literature on histone O-GlcNAcylation shows that there is currently no evidence that histone proteins would be modified at a consensus site, that many of the residues reported to be modified are inaccessible to Ogt in the context of a nucleosome, and that functional tests to substantiate a role of histone O-GlcNAcylation are largely missing. Future studies will undoubtedly provide a more comprehensive understanding of the physiological functions in the nucleus that are controlled by Ogt and thus help to explain why this enzyme is essential for the viability of mammalian cells.
